# Morbilliform Eruption After Administration of Second Dose of Oxford/AstraZeneca Vaccine

**DOI:** 10.7759/cureus.24649

**Published:** 2022-05-01

**Authors:** Norah S Alhammad, Heba H Milibary, Razan R Baghdadi, Toleen M Alawadi, Rawan E Hudairy

**Affiliations:** 1 Dermatology, King Fahad Armed Forces Hospital, Jeddah, SAU; 2 Dermatology, King Abdulaziz University Faculty of Medicine, Jeddah, SAU; 3 Dermatology, King Abdulaziz General Hospital, Makkah, SAU; 4 Anatomic Pathology, King Faisal Specialist Hospital and Research Centre, Jeddah, SAU

**Keywords:** covid- 19 case report, morbilliform, covid 19 vaccine complication, covid-19 vaccine, oxford-astrazeneca vaccine, morbilliform drug eruption, covid 19

## Abstract

Morbilliform eruption typically implies a maculopapular rash of acute onset. Drugs are the predominant cause of this cutaneous reaction in adults, followed by infectious exanthems and some rheumatological diseases. In this article, we report on the clinical and histopathological features of generalized pruritic morbilliform eruption in a 28-year-old female following her second dose of Oxford/AstraZeneca COVID-19 vaccine. The reaction started 12 hours after receiving the vaccine with no other identifiable cause.

The patient had no improvement with IV antihistamine received in the emergency department. Afterward, she showed marked improvement after receiving a short course of oral corticosteroids along with topical corticosteroid and oral antihistamine. To the best of our knowledge, we hypothesize that the basic immunological mechanism is the cause behind COVID-19-vaccine-related morbilliform eruption. Therefore, physicians should be aware of the possible adverse reactions associated with COVID-19 vaccines, such as morbilliform eruptions and other cutaneous manifestations.

## Introduction

Coronavirus disease 2019 (COVID-19) has affected more than 500 million people worldwide, besides approximately 6 million deaths occurred with the ongoing catastrophic pandemic [1], which led to joining efforts in the search of rapid invention and approval for vaccines that could reduce panic, mortality and socio-economic consequences. Since December 2020 numerous potent vaccines against COVID-19 have been developed and permitted in record time [2]. Oxford/AstraZeneca vaccine was authorized by the European Medicines Agency (EMA) for use across the European Union (EU) following endorsement by the European Commission on 29 January 2021 [3]. It is a monovalent vaccine developed with a recombinant adenoviral vector from chimpanzees encoding the S glycoprotein of SARS-CoV-2, which stimulates cellular immune response after administration to the human body [4]. Regarding side effects, cutaneous adverse drug reactions (ADRs) seem to be common events in the course of COVID-19 vaccines. Oxford-AstraZeneca cutaneous ADRs are mainly mild to moderate in severity. No serious adverse reactions happened during the trials [5]. Herein, we report a case of generalized pruritic morbilliform eruption that developed after receiving the second dose of Oxford/AstraZeneca COVID-19 vaccine.

## Case presentation

A 28-year-old female patient, medically free, presented with a two-day history of generalized pruritic maculopapular rash over her face and neck then spread to involve the trunk, bilateral upper extremities and palms, bilateral thighs, preserving bilateral distal lower extremities and soles of the foot. It started 12 hours after receiving the second dose of AstraZeneca COVID-19 vaccine. There was no history of any rash after receiving the first dose of the same vaccine. Rash was not associated with fever, cough, joint pain, headache, fatigue or malaise, and other systemic review was unremarkable. There were no similar complaints from the family members, no previous history of COVID-19 infection, allergy to food or medications and no history of previous blood transfusion or surgery.

**Figure 1:**
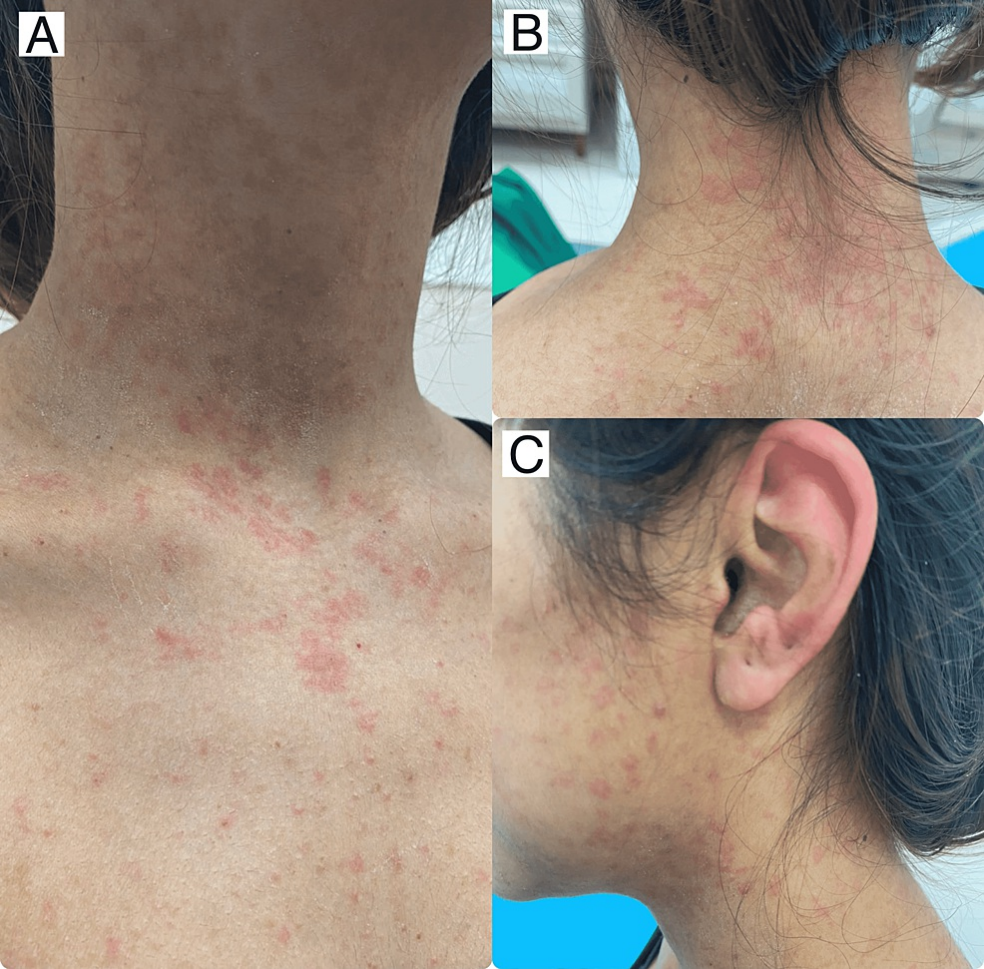
There are multiple erythematous non-scaly macules and papules over the chest (A), neck (B) and face (C), some coalescing into patches. Note the involvement of ear pinna with erythematous non-scaly patches.

**Figure 2:**
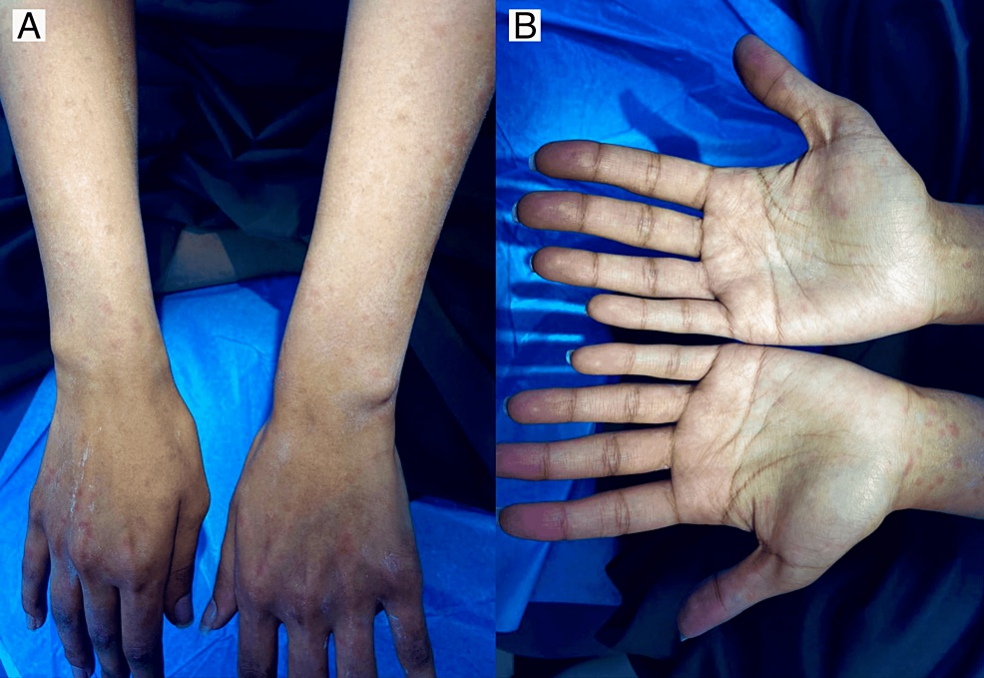
There are multiple erythematous non-scaly macules and papules over bilateral upper arms, dorsum of hands (A) and bilateral palms (B).

**Figure 3:**
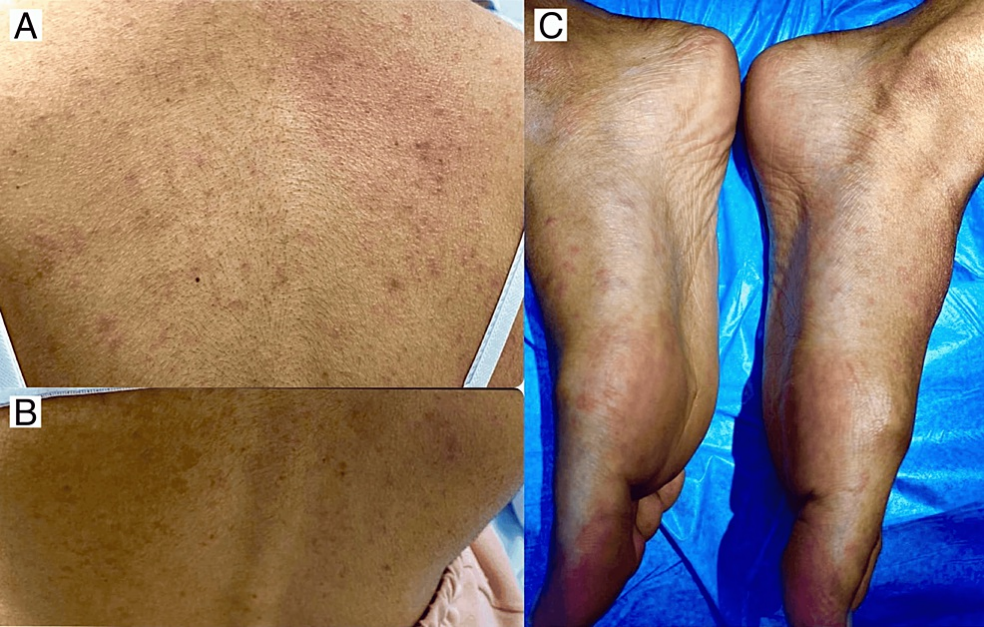
There are multiple erythematous non-scaly macules and papules over the back (A, B) and bilateral soles (C).

The patient went to the emergency department and received IV antihistamine (one dose of diphenhydramine 50 mg/ml) along with oral antihistamine (10 mg loratadine once daily) but there was no improvement, and the lesions remained the same for 24 hours. Skin examination revealed a generalized eruption of erythematous non-scaly maculopapular rash over the face, neck, trunk, bilateral upper and lower extremities, bilateral palms and soles (Figures 1-3). There was no mucosal or nail involvement.

**Figure 4:**
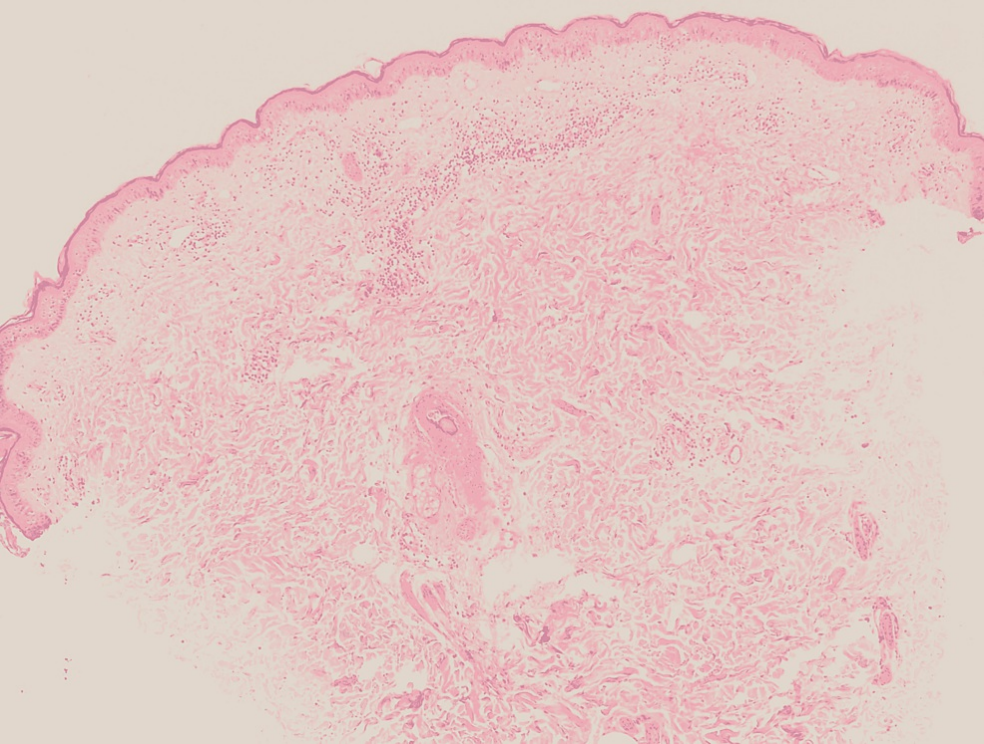
Histopathological features Low power view showing skin punch biopsy, exhibits vacuolar degeneration of the basal layer and perivascular infiltrate of the superficial plexus.

**Figure 5:**
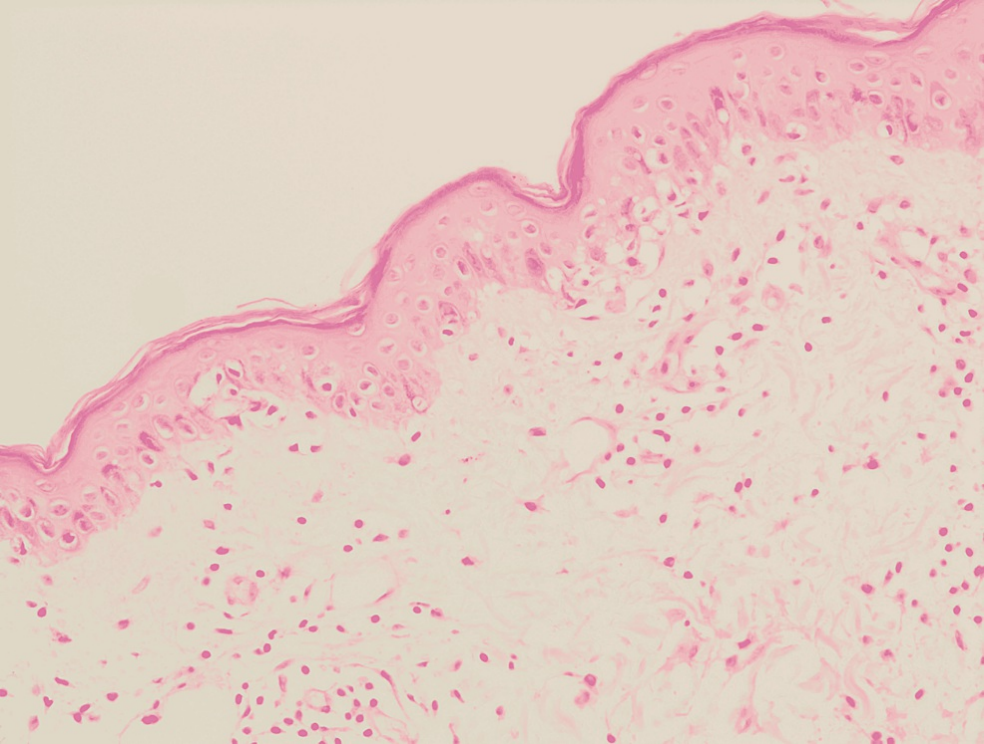
Histopathological features Intermediate power showing vacuolization of the basal layer with occasional lymphocytes exocytosis, with perivascular infiltrate of the superficial plexus, predominately of lymphocytes.

**Figure 6:**
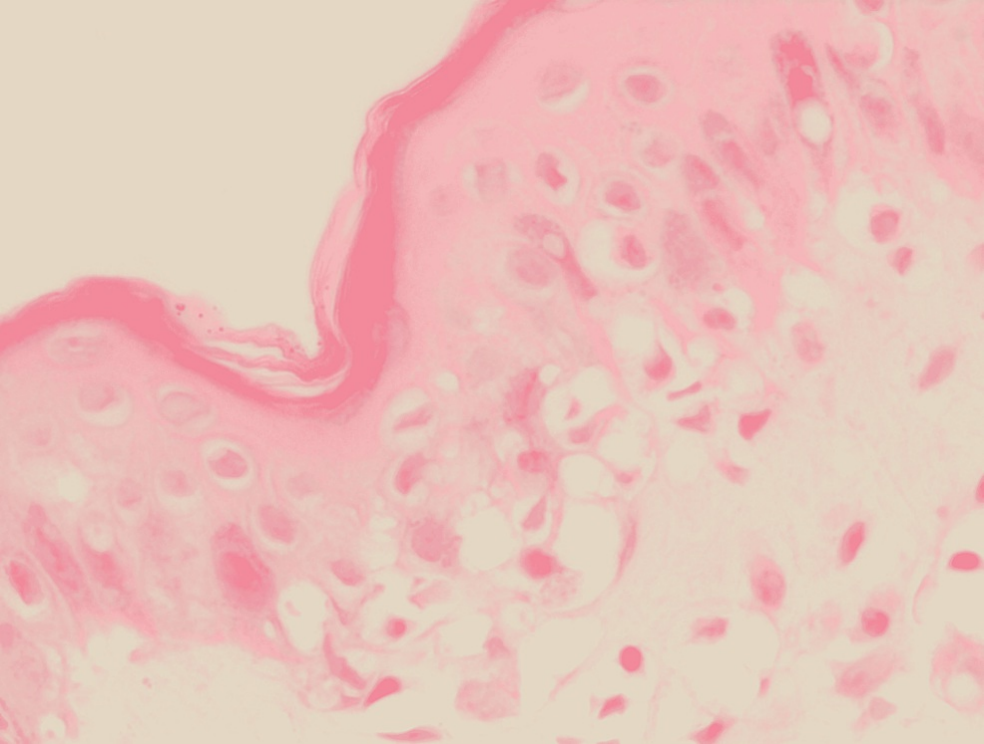
Histopathological features High power view demonstrates interface dermatitis in the form of vacuolar degeneration of the basal layer with occasional lymphocytes exocytosis.

Histopathology of skin biopsy using a 3-mm punch pen taken from the patient’s left arm after signing consent showed epidermal hyperkeratosis, vacuolar degeneration of the basal layer, exocytosis and mild spongiosis. The dermis exhibited mild perivascular lymphocytic infiltrate in the superficial plexus and rare interstitial eosinophils. There was no evidence of acanthosis and extravasated red blood cells (RBC) (Figures 4-6).

We prescribed the patient a short course of oral corticosteroid (prednisolone 20 mg tapered over one week), oral desloratadine 5 mg once daily for seven days and topical mometasone cream twice per day for seven days. Follow-up after one week showed marked improvement of the lesions with no post-inflammatory changes.

## Discussion

In a nationwide study about cutaneous adverse reactions of COVID-19 vaccines, out of 405 reported cases who received either Oxford-AstraZeneca, Pfizer or Moderna vaccines, the most reported reactions were local injection site reactions like erythema and swelling followed by urticaria and morbilliform rash respectively. The mean onset time was 5.1 days after vaccination and the mean duration was 12.2 days. However, most reactions were mild-moderate in severity and self-limiting, but some required treatment mostly with topical/systemic steroids or oral antihistamines [6]. On the other hand, there were few reported cases of flare of psoriasis [7], Erythema nodosum, zoster duplex reactivation and pityriasis rosea as possible cutaneous adverse effects of Oxford-AstraZeneca COVID-19 vaccine [8].

In our case report, the patient developed morbilliform rash secondary to Oxford AstraZeneca vaccine, that interestingly developed 12 hours after receiving the second dose, however, no cutaneous reaction was reported after receiving the first dose. All lesions resolved after a short course of oral corticosteroids. Similar morbilliform rash has been reported following the first and second dose of Pfizer-BioNTech COVID-19 mRNA vaccine in a case report from the United States with lesions resolved spontaneously within 24 hours. The development of cutaneous reaction primarily to the second dose may be explained by the process of immune-mediated response [9].

Moreover, many factors might be associated with the development of morbilliform drug eruption. Sociodemographic factors such as age, gender, and living conditions might increase the risk of this skin disease. It occurs more commonly in the elderly, inpatients, females, and the immunocompromised. In line with available evidence, our patient was a healthy young immunocompetent female.

In addition, the cycle of transferring the vaccine from its manufacturing company until it reached the consumer might be influenced by many factors such as demographic areas. For example, the weather in Saudi Arabia is mostly hot during the year which might negatively affect the efficacy of the vaccine [10].

With that being said, further research is required to find and describe cutaneous adverse reactions in order to enhance the level of knowledge about it, which may help healthcare professionals to adjust the current guidelines depending on the new findings and to reassure/council their patients accordingly.

## Conclusions

We have described a case of new-onset cutaneous reaction post second dose of Oxford/AstraZeneca COVID-19 vaccine, which was mostly mild or moderate in severity. Fortunately, medical treatment with steroids is usually successful for the management of this condition. Even though these adverse events are relevant, they should not delay patients from receiving necessary doses to fight against the virus, as the benefits overweigh the risks in this ongoing pandemic and the emerging features of SARS-CoV-2 variants.
